# Intraoperative Cognitive Mapping Tasks for Direct Electrical Stimulation in Clinical and Neuroscientific Contexts

**DOI:** 10.3389/fnhum.2021.612891

**Published:** 2021-03-02

**Authors:** Linghao Bu, Junfeng Lu, Jie Zhang, Jinsong Wu

**Affiliations:** ^1^Department of Neurosurgery, Huashan Hospital, Shanghai Medical College, Fudan University, Shanghai, China; ^2^Brain Function Laboratory, Neurosurgical Institute of Fudan University, Shanghai, China; ^3^Zhangjiang Lab, Institute of Brain-Intelligence Technology, Shanghai, China; ^4^Shanghai Key Laboratory of Brain Function and Restoration and Neural Regeneration, Shanghai, China; ^5^Shanghai Clinical Medical Center of Neurosurgery, Shanghai, China

**Keywords:** direct electrical stimulation, cognitive function, awake surgery, neuroscience, neurophysiology

## Abstract

Direct electrical stimulation (DES) has been widely applied in both guidance of lesion resection and scientific research; however, the design and selection of intraoperative cognitive mapping tasks have not been updated in a very long time. We introduce updated mapping tasks for language and non-language functions and provide recommendations for optimal design and selection of intraoperative mapping tasks. In addition, with DES becoming more critical in current neuroscientific research, a task design that has not been widely used in DES yet (subtraction and conjunction paradigms) was introduced for more delicate mapping of brain functions especially for research purposes. We also illustrate the importance of designing a common task series for DES and other non-invasive mapping techniques. This review gives practical updated guidelines for advanced application of DES in clinical and neuroscientific research.

## Introduction

The persevering efforts of Penfield (Penfield and Boldrey, 1937; Penfield, 1958), Ojemann (Ojemann et al., 1989), Duffau (Tate et al., 2014; Rech et al., 2019), Berger (Sanai et al., 2008), and other neurosurgeons (Roux et al., 2003, 2004; Démonet et al., 2011; Wu et al., 2011) in awake surgery coupled with direct electrical stimulation (DES) led to the wide application of DES in identifying the “eloquent area” before lesion resection due to its high effectiveness and reliability. Accumulating evidence lent support to the importance of this technique, which aided in achieving more extensive resection with lower permanent neurological deficits (Sacko et al., 2011; De Witt Hamer et al., 2012; Gerritsen et al., 2019). Apart from clinical application, DES also provides a tremendous opportunity for neurosurgeons to step into neuroscientific research. Unlike traditional lesion studies, DES disturbs temporarily and reversibly the neural activity in a limited brain area or neural network. This method largely avoids the influence of functional reorganization after focal brain damage and provides a causal link between neural substrates and cognitive functions (Desmurget et al., 2013; Vaidya et al., 2019). Following in the footsteps of Penfield’s famous cortical homunculus research, other higher-order cognitive functions have been investigated using DES in the past decades pushing forward the frontiers of neuroscience (Sanai et al., 2008; Tate et al., 2014; Rech et al., 2019).

With the increasing popularity of DES in both clinical and scientific research, multiple teams have developed their own intraoperative cognitive mapping tasks, and the brain functions they mapped extend from sensorimotor to language and even non-language functions (Duffau, 2010). However, the details of task design and selection were rarely reported, leading to potential heterogeneity between institutions in mapping results and the efficacy of electrical stimulation. Therefore, a practical and standardized protocol for intraoperative task design and selection is needed in awake surgery. Moreover, with the frontier of neuroscience expanding, traditional tasks no longer fulfill the needs of current research; thus, the design of more complicated mapping tasks has become an important issue.

In this paper, we first summarize the electrical stimulation parameters (current intensity, stimulation duration, stimulation onset) used in DES. Next, we introduce popular tasks for the mapping of cognitive function and further propose a protocol for task selection to guide lesion resection. In addition, recent neuroscientific findings based on DES were reviewed, and an optimal way to design a delicate mapping task series for research purposes was demonstrated. With the increasing popularity of multimodal fusion in both clinical and scientific research, the importance of common task series for different mapping techniques was proposed.

## Electrical Stimulation Parameters

Current intensity is one of the most important parameters that affects the sensitivity and specificity of DES. During cortical mapping, a bipolar electrode is used to deliver 1 ms (0.5 ms for anodal phase and another 0.5 ms for cathodal phase) biphasic square waves at 60 Hz, which facilitates the excitement of neural cells parallel to the bipolar axis. There are two main mapping strategies with distinct current intensities. Some researchers adopt a “negative mapping strategy” in which the amplitude for each cortical site is increased from 2 mA to a predetermined threshold (usually 6 mA) or until an after discharge is evoked (Sanai et al., 2008; Sarubbo et al., 2013). During cognitive mapping, the current intensity was 0.5–1 mA lower than the intensity determined in motor mapping (Sanai et al., 2008; Sarubbo et al., 2013; Hervey-Jumper et al., 2015). After brain mapping, tumor resection was guided by the “negative sites” that referred to the cortical areas without stimulation-induced motor or language function. Therefore, a minimal cortical exposure was allowed in this strategy. Another mapping strategy, which was used in our institute, focused more on the identification of positive cortical sites. The stimulation also began with the localization of primary sensory or motor area, but the current intensity increased gradually until the detection of sensory or motor changes. This intensity was then used for the cognitive mapping. Even though a larger cortical exposure was needed, more positive sites could be identified with this strategy, which made it more popular in current neuroscientific researches (Duffau et al., 2004; Khan et al., 2014; Tate et al., 2014; Sarubbo et al., 2020). Since the frequency was slightly different among institutes, the charge density/phase is a reliable parameter to describe the current intensity (Gordon et al., 1990). This parameter was widely used in animal studies (Agnew et al., 1983; Agnew and McCreery, 1987) or extraoperative stimulation (Elisevich et al., 2006; Cogan et al., 2016); however, it was rarely used in studies about awake surgery. Gordon et al. (1990) investigated the histological change within the proximity of electrode in the human brain, but the charge density/phase was lower than that commonly applied during intraoperative DES and the influence of electrical stimulation remained unknown. Therefore, further studies are needed to settle the safe threshold of charge density/phase.

Although numerous studies have used DES to reveal the functions of white matter tracts, rarely has a study provided detailed analysis of the current threshold of subcortical mapping (Bello et al., 2007; Duffau et al., 2008; Zemmoura et al., 2015; Puglisi et al., 2019). Most stated that the same intensity for cortical mapping was also used for subcortical mapping (Bello et al., 2007; Fujii et al., 2015; Puglisi et al., 2019). Even though the underlying neurophysiological mechanisms remains unknown and no clinical research is available to verify the efficacy, this strategy is now the only practical way to set current intensity for subcortical mapping and therefore is also the strategy adopted by our institute.

Another stimulation parameter needed to consider is the time course. Normally, the stimulation lasts 1 s for language and other non-language mapping and is applied slightly earlier than stimulus onset. More recently, Morshed et al. (2020) proposed that prolonged stimulation duration resulted in higher sensitivity of DES. With the identification of time course of human cognitive activities, other studies have also attempted to change the temporal relation between electrical stimulation and stimulus to interfere a specific physiological stage (Nakai et al., 2019; Morshed et al., 2020). Further studies are needed on these new mapping protocols.

## Intraoperative Tasks in Clinical Context

### Language Tasks

Currently, a majority of the DES carried out clinically are performed to locate language areas in the dominant hemisphere. A set of common language mapping tasks (counting, picture naming, reading) has been used since the era of Penfield (Penfield and Boldrey, 1937; Penfield, 1958), with the detailed design and selection of these tasks varying between institutes and countries.

#### Number Counting

Counting is an automatic speech task that is widely used in language assessment especially in patients who cannot complete complex cognitive tasks (Bookheimer et al., 2000). During language mapping, number counting is carried out first to identify cortical and subcortical structures related to speech output (Fernandez Coello et al., 2013; Mandonnet et al., 2017). The patients are asked to count from 1 to 50, and an interruption without movement (i.e., speech arrest) can be induced when stimulating the ventral premotor cortex (Sanai et al., 2008; Tate et al., 2014; Wu et al., 2015; Sarubbo et al., 2020), inferior frontal gyrus (Sanai et al., 2008; Wu et al., 2015), or area 55b (Rech et al., 2019), as well as white matter fibers including frontal aslant tract (Kinoshita et al., 2015) and arcuate fasciculus (Duffau, 2015; Sarubbo et al., 2020). Dysarthria is another disruption that is elicited during counting. Dysarthria-related sites are distributed within the lateral precentral and postcentral gyrus over the underlying white matter tracts (e.g., superior longitudinal fasciculus-III) (Tate et al., 2014; Duffau, 2015; Sarubbo et al., 2020) that participate in the articulatory network.

#### Picture Naming

Picture naming is one of the most widely used tasks in language mapping. Most institutions follow the protocol that was first used byPenfield and Boldrey (1937) and Penfield (1958), and further described in detail by Ojemann and Mateer (1979). In this task, pictures of common objects are presented to patients one by one, and each is shown for 4 s. Patients need to name the object with a carrier phrase “This is a …” as soon as possible.

**FIGURE 1:**
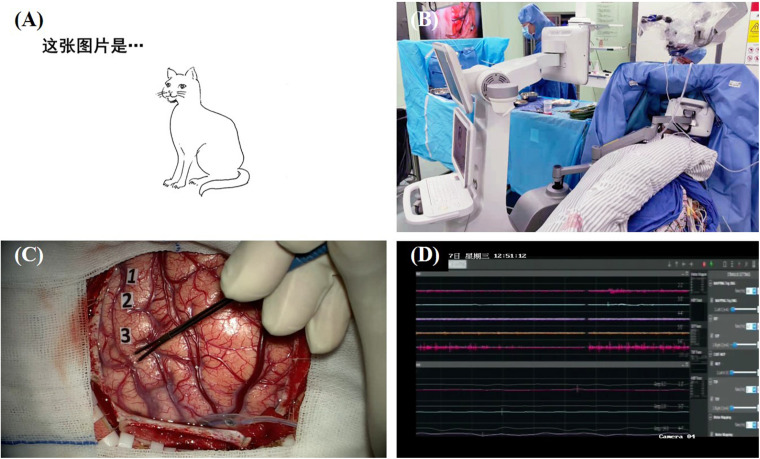
Illustration of direct electrical mapping with picture naming task. **(A)** A series of black-and-white line drawing of a common object is used as stimulating materials; **(B)** a specially designed machine is used to present the stimulus with a moveable screen [*more details about this machine on our forthcoming article (Hameed et al., 2021)*]; **(C)** neurosurgeon applies electrical stimulations with a bipolar electrode and uses sterile tags to mark the positive sites; **(D)** electrophysiological monitoring is used to record after-discharge activity.

One major advantage of the picture naming task is that it is a “multifunctional” task that can evaluate multiple brain functions including visual recognition, conceptual formation, lexical retrieval, and the final speech output (Gleichgerrcht et al., 2015). Errors can be induced by the disruption of any single stage in naming processing. The most common language interference found in this task is anomia, which is defined as the DES-induced phenomenon in which the patient is able to speak out the carrier phrase but unable to name the object (Sanai et al., 2008) or name with incorrect words. Other errors [e.g., perseveration (Duffau et al., 2005, 2008; Gil Robles et al., 2005; Khan et al., 2014; Mandonnet et al., 2019b), hesitation (Hamberger et al., 2005), and tip-of-the-tongue (Hamberger et al., 2005)] are also reported in the literature. In addition, since speech output can also be assessed by this task, some researchers map speech arrest during picture naming instead of counting (Petrovich Brennan et al., 2007). DES-induced speech arrest can be distinguished from anomia through the inability of saying the carrier phase. Figure 1 shows an illustrative case of direct electrical mapping with picture naming task.

Although task protocol and the definition of DES-induced errors are relatively consistent, stimulating materials vary among different institutes, and only a few studies report the source of the pictures they used. Compared to image sets found randomly from the Internet, usage of batteries and image sets with normative data available is more recommended. Duffau et al. (2005); Mandonnet et al. (2017, 2019b), and Rech et al. (2019) select pictures from DO 80, which comprises of 80 black-and-white pictures with variables such as frequency, familiarity, and age of acquisition controlled. Some teams advocate the application of the Boston Naming test, which contains 60 line drawings with decreasing frequency. However, both our experience and previous literature (Chen et al., 2014) has revealed that this test contains several objects with relatively low frequency in Chinese (e.g., igloo, harp), which causes a higher probability of false positive. Therefore, cultural adjustment is needed in intraoperative task design. In our institute, the Snodgrass and Vanderwart picture set (Snodgrass and Vanderwart, 1980) is adopted, since the test contains 260 easily recognizable drawings of common objects from different categories and, more importantly, has norms for Mandarin Chinese available (Liu et al., 2011). For teams from other countries, the International Picture-Naming Project (IPNP) provides norms in seven different languages (American English, German, Mexican Spanish, Italian, Bulgarian, Hungarian, and Chinese) for a corpus of 520 drawings including those from the Snodgrass and Vanderwart picture set. Thus, this picture set showed a distinct advantage in language mapping.

In addition, another important point is the selection of drawings. If the difficulty of a naming task is too high, some intraoperative naming errors may not be directly caused by electrical stimulation and further lead to false positive readings. Thus, Little and Friedman (2004) proposed that patients should be trained preoperatively, and pictures that could not be named by most patients should be removed from the intraoperative task. However, no quantitative parameters like frequency, age of acquisition, and familiarity are used in their selection criteria, which deteriorates its practical value for other institutes. Previous psycholinguistic research have demonstrated that familiarity and age of acquisition are two major predictors for naming latencies (Weekes et al., 2007; Liu et al., 2011); thus, we recommend that only pictures with high concept familiarity and low age of acquisition should be included into intraoperative tasks to ensure the reliability of language mapping. Further studies are still needed to determine the cutoff of these parameters in our new selection criteria.

#### Other Language Mapping Tasks

Besides these two major tasks (counting and picture naming), there are several other language mapping tasks reported in the literature. Several studies demonstrated that stimulating the posterior temporal lobe (Sanai et al., 2008) and inferior parietal lobule (Roux et al., 2004; Sanai et al., 2008; Wu et al., 2015) could induce alexia in word reading task. Tasks such as repetition (Fernandez Coello et al., 2013; Mandonnet et al., 2017; Sarubbo et al., 2020) and writing (Roux et al., 2003; Morrison et al., 2016) are also performed according to the tumor location (e.g., applying repetition task to identify arcuate fasciculus; Moritz-Gasser and Duffau, 2013). Moreover, for multilingual patients, a sentence translation task could be used to protect their function of switching between multiple languages.

In a recent review, Rofes and Miceli (2014) emphasized the importance of tasks with verbs and sentences in language mapping because the daily language activity poses far more demands on the cognitive network than simple automatic speech and picture naming tasks applied in awake surgery. More sophisticated tasks (e.g., auditory naming, sentence comprehension, completion, and reading) could be designed and added into language mapping procedure. These tasks could engage speech output, as well as conceptual formation and lexical retrieval in picture naming tasks, and in addition, higher level language processing such as syntax could also be tackled. Although these tasks are currently rarely applied in clinical setting due to their complexity, these tasks shed light on the sophisticated human language activity.

### Non-language Tasks

Despite the increasing application of DES by neurosurgeons in the past few decades (Sanai et al., 2008; De Witt Hamer et al., 2012; Tate et al., 2014; Rech et al., 2019), a majority of cases enrolled are those with lesions within or near the language areas in dominant hemisphere, and only language tasks are applied routinely during surgery. Recently, studies have started focusing on other non-language functions that also have a strong relation with postoperative quality of life (Duffau et al., 2004; Duffau, 2010; Gras-Combe et al., 2012; Fox et al., 2020).

#### Vision

Visual processing is another important neurological ability used for perceiving the surrounding environment. Previous studies based on non-invasive methods such as functional MRI (fMRI), lesion studies, and electroencephalography have proposed a dual stream network of visual processing. Like language deficits, disruptions in this network may lead to failure of sensory awareness of visual stimulus and interfere with daily activities. In order to protect such a critical function, tasks are added to detect the structures related to visual processing.

Permanent visual field deficit due to disruption of optic radiation is a common postoperative complication of patients with lesions in temporal or occipital lobe. Despite the application of multimodal neuroimaging in visual protection, direct mapping of optic radiation was not achieved by these non-invasive methods. Duffau et al. (2004) first reported that electrical stimulation of optic radiations induced visual disorder during picture naming task in a patient with lesions invading the temporal lobe and the temporo-occipital junction. An intraoperative visual task was later introduced in Gras-Combe et al. (2012). In this task, patients are asked to name the two pictures located diagonally on the screen with their eyes fixed on the center. They reported that all the 14 patients experienced visual symptoms during awake surgery; thus, optic radiations were identified and protected with only one patient having permanent postoperative hemianopia. Recently, an integrated method of visual pathway tracking using both diffusion tensor imaging and DES was reported, indicating the potential advantages of multimodal brain mapping (Mazerand et al., 2017).

Another deficit in visual processing is spatial neglect. Unlike visual field deficit, spatial neglect is an attentional deficit in which the patient has an intact visual field but ignores part of the visual space. Even though this phenomenon has gained the attention of neurologists and there are extensive number of assessments available, few studies report on a method to protect visuospatial function during neurosurgery. The main reason for this is that visuospatial function is related to non-dominant hemisphere whose cognitive role has long been ignored. Some pioneers have proposed the line bisection task, which could be used to map visuospatial function. In this task, patients are asked to mark the midpoint of a line with a pen or finger. Some researchers have successfully identified that spatial neglect in line bisection task could be induced when electrically stimulating the inferior parietal lobule and the posterior temporal lobe (Thiebaut de Schotten et al., 2005; Démonet et al., 2011; Velasquez et al., 2018), which is consistent with fMRI results. Démonet et al. (2011) reported that no postoperative spatial neglect was found in all 20 cases whose positive sites of line bisection errors were spared, indicating the clinical value of line bisection task. Other neuropsychological tests including clock test and judgment of line orientation for visuospatial function could also be used in awake surgery. Compared to the line bisection task, these tasks are easier to complete during surgery since no hand movement is needed. However, these new tasks have not been widely applied yet, and further investigation is needed to illustrate the reliability of these tasks in DES for minimizing the postoperative spatial neglect.

#### Other Higher Cognitive Functions

Compared to visual field and visuospatial function, other higher cognitive functions (e.g., calculation, working memory, music) have received lesser attention during surgery. However, these functions are also closely related to patients’ quality of life. For example, arithmetic processing is an indispensable brain function for a mass of daily activities, and recently, a few teams have begun using calculation task to prevent permeant acalculia (Duffau et al., 2002; Della Puppa et al., 2013; Della Puppa et al., 2015; Matsuda et al., 2019). Another important issue in mapping higher cognitive functions is that patients have differing demands of their postoperative functions based on their jobs and lifestyles. We have previously reported on a music student with glioma in Broca’s area (Zhang et al., 2013). In order to protect her musical ability, two music tasks including humming a popular Chinese song and reading a piece of musical note were performed after routine language mapping. During surgery, two music interference sites were identified and spared. Postoperative evaluation showed that her music function had surprisingly improved slightly relative to the preoperative level.

In addition to the cognitive functions mentioned above, studies have also attempted to locate domain-general cognitive networks involved in emotion and working memory. Thus, more complex tasks like emotion (Gordon et al., 1996; Fox et al., 2020), Stroop test (Wager et al., 2013; Puglisi et al., 2019), and N-back test (Motomura et al., 2018, 2019) were introduced into awake surgery. Fox et al. (2020) showed that stimulation in the limbic system could elicit different emotion responses (e.g., joy, nervousness). For the N-back task, errors could be elicited when stimulating the dorsolateral prefrontal cortex (Motomura et al., 2018, 2019). It is important to note that in most studies (Démonet et al., 2011; Fox et al., 2020), these higher cognitive function tasks are performed solely for scientific purpose instead of function preservation, and the identified neural substrates were resected even though positive sites existed. Therefore, further studies, especially those with large cohorts, are needed to settle their practicability in clinical contexts.

### Optimal Selection of Intraoperative Tasks

Varying cognitive mapping tasks available for different cognitive domains are accumulating with the increasing application of DES. However, the maximum duration that patients can stay conscious and complete mapping tasks is about 1 h based on our and others experience (Mandonnet et al., 2019a), which means that tasks used in surgery is tightly limited. Under these circumstances, tasks that integrate multiple cognitive domains are recommended more since several cerebral functions can be evaluated simultaneously.

During tasks selection, the first thing to take into account is the patient’s basic information, including job, hobbies, lifestyles, and, more importantly, their preoperative cognitive function. Thus, a set of detailed neuropsychological assessments should be performed to set a baseline for each patient. In our institute, every patient is assessed pre- and postoperatively using a series of comprehensive batteries, including a questionnaire for personal information, the Edinburgh Handedness Inventory for handedness, the Mini-Mental State Examination for potential cognitive impairments, as well as the Boston Naming Test and the Aphasia Battery of Chinese for language function. Calculation and visuospatial function are also evaluated in the Aphasia Battery of Chinese. Based on the assessment results of different cognitive functions, tasks are chosen based on the preoperatively intact or mildly compromised functions deserving more attention.

**FIGURE 2:**
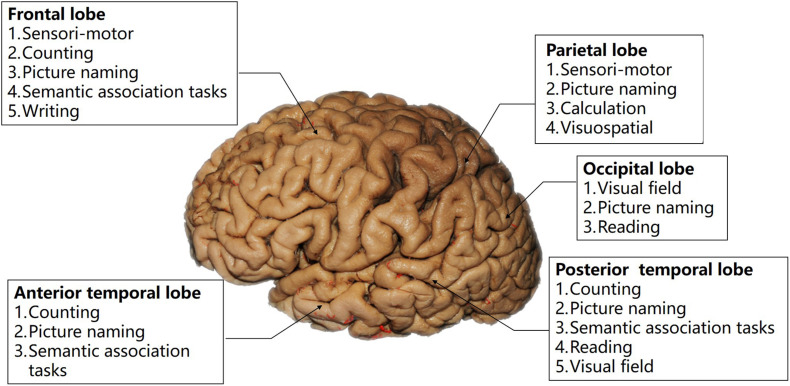
Illustration of the relation between intraoperative tasks and cortical regions.

**FIGURE 3:**
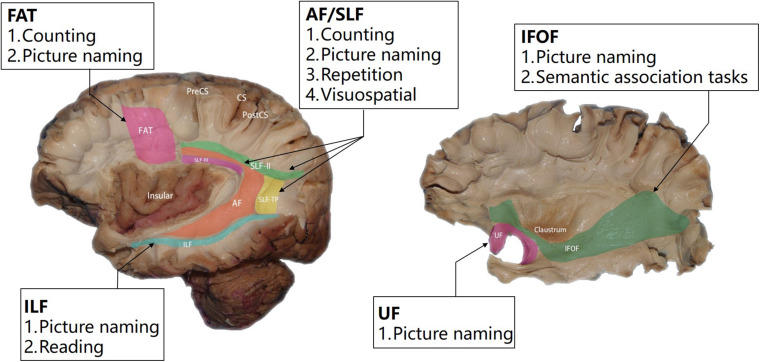
Illustration of the relation between intraoperative tasks and major white matter tracts.

Characteristics of a tumor, especially its location, is another important factor in task selection. Figure 2 illustrates the tasks recommended for lesions in different regions. In addition, the role of white matter tracts in cognitive function has been confirmed by multiple studies and Fernandez Coello et al. (2013) proposed that plasticity of white matter tracts made them more crucial in brain function protection. In neurosurgery, these tracts mark the boundary of lesion resection; thus, applying subcortical electrical stimulation to identify these fiber tracts is as important as cortical mapping. Based on the available neuroscientific research results, we present our proposed intraoperative tasks for tumors invading different white mater tracts in Figure 3.

Table 1 summarizes common language and non-language tasks that we recommend for clinical practice. Information including hemisphere, lesion location, task design, and responses are also given.

**TABLE 1:** Summary of recommended language and non-language tasks.

Cognitive functions		Tasks	Tumor location			Task design		Responses
			Hemis-phere	Cortical regions	WM tracts	Stimulus	Carrier phrase	
Language	Standard	Number counting	L	Perisylvian region	FAT, AF	NA	NA	Speech arrest, dysarthria
		Picture naming	L	Perisylvian region	SLF, AF, IFOF, UF, ILF	Line drawings from *Snodgrass and Vanderwart picture set*	This is a …	Speech arrest, dysarthria, anomia, phonological/semantic paraphasia
	Optional	Reading	L	PTL, IPL	IFOF, ILF	Words/Sentences	This word/sentence is …	Alexia
		Repetition	L	IPL	AF, SLF	Words/Sentences	NA	Repetition deficit
Non-language		Vision	L, R	TPOJ	Optic radiations	Two line drawings located diagonally on the screen	These two pictures are …	Blurred vision, phosphenes, visual field deficit
		Line bisection	R	IPL	SLF	A horizontal line	NA	Spatial neglect
		Calculation	L, R	IPL	SLF	1-digit addition with 1 operand	NA	Hesitation, no answer, incorrect answer

**PTL, posterior temporal lobe; IPL, inferior parietal lobule; TPOJ, temporo-parieto-occipital junction; FAT, frontal aslant tract; AF, arcuate fasciculus; SLF, superior longitudinal fasciculus; IFOF, inferior frontal occipital fasciculus; ILF, inferior longitudinal fasciculus; UF, uncinate fasciculus.**

## Des and Intraoperative Tasks in Neuroscientific Research

In addition to its application as a clinical tool, DES is currently a popular method in neuroscientific research due to its ability to probe the causal relation between brain functions and structures.

As mentioned above, DES is mostly performed to identify language areas; therefore, most significant results have been obtained in neurolinguistic research. In the past 20 years, tasks such as counting, picture naming, and reading have been used by different teams to construct the probabilistic maps of language areas. A preliminary consensus had been achieved that key regions for language processing include the pars triangularis, pars opercularis, and ventral precentral gyrus in frontal lobe, as well as the posterior part of temporal lobe (Sanai et al., 2008; Wu et al., 2015; Sarubbo et al., 2020). Language-related white matter tracts were also revealed for the first time using subcortical stimulation, demonstrating the structural connectivity between key language areas. Furthermore, Duffau (2015) proposed a language processing model, which illustrates the anatomical basis of dual stream model of Hickok and Poeppel (Hickok and Poeppel, 2007; Hickok, 2012).

While the general framework of language processing has been constructed, there are still controversies on the exact role that a certain brain region play. Therefore, current neurolinguistic studies focus more on a specific stage in language processing rather than the whole stream. However, traditional tasks, like picture naming, engage plenty of processing stages, and the interruption of each stage could cause dysfunctions. In most studies, despite the generation of spatial map of anomia, it is nearly impossible to identify which stage in language processing was blocked in stimulation-induced anomia. Thus, the functional role of each structure is illustrated by evidence from other modality [e.g., fMRI, electrocorticography (ECoG), lesion studies] rather than direct evidence from DES. To address this issue, some researchers adopted the cognitive subtraction and conjunction paradigms in fMRI studies. These two paradigms usually contain a series of tasks, so, as their names suggest, the subtraction paradigm aims to locate the different processing stage between tasks and the conjunction paradigm is designed to locate the shared one (Price and Friston, 1997). Forseth et al. (2018) combined these two paradigms and compared the disruption of positive sites for a series of tasks including picture naming, naming to definition, sentence repetition, and sensorimotor effects. For the first time, a solid evidence derived from DES was provided to support the role of middle fusiform gyrus as the lexical semantic hub. Another study from Lee and his colleagues also applied the same paradigm and successfully demonstrated that the morphological characteristic in syntax is attributed to a small region within the posterior part of superior temporal gyrus (Lee et al., 2018). Even though the application of this task design strategy is still in its initial stage, an increasing amount of literature using this strategy could be expected since there are abundant task series available for adaptation in previous fMRI studies.

## Common Tasks for DES and Other Modalities

Despite its irreplaceable role as the sole reliable method to identify the eloquent neural substrates during surgery, DES, as an invasive mapping method, cannot be applied pre- and postoperatively. Except for the rare studies based on repeated brain mapping in cases with glioma recurrence (Krieg et al., 2014; Southwell et al., 2016), findings on brain plasticity are mainly derived from non-invasive techniques including fMRI, transcranial magnetic stimulation (TMS), and positron emission tomography (PET). Several case reports have tried to investigate the perioperative reorganization of cognitive networks with both DES and other techniques (Krieg et al., 2014; Spena et al., 2015). However, the task variation between techniques in studies affects the reliability of the findings. Therefore, a common task series that could be applied for different modalities is needed for the direct comparison of eloquent structures pre-, intra-, and postoperatively. In our institute, a set of language tasks, including counting, reading, and picture naming, is used in task-fMRI, DES, as well TMS for perioperative language mapping. More tasks for other cognitive domains could also be added into this common task series in our future work. This common task series can be used to not only investigate brain plasticity but also compare the reliability of DES and other non-invasive techniques in brain mapping.

## Conclusion and Perspective

In spite of the increasing number of institutes applying DES in the clinical context, the design and selection of mapping tasks vary among institutions. In this review, we proposed a practical method for the optimization of existing mapping tasks and personalized selection of tasks based on patients’ situation. Such a universal principle in task design and selection may provide guidance for other teams and further eliminate the heterogeneity caused by tasks difference between institutions. Additionally, to match the advances in neuroscientific research, direction of task design (i.e., subtraction and conjunction paradigms) for the next era of DES studies is discussed, and the importance of combing DES and other techniques is also illustrated.

## Author Contributions

JW and LB were responsible for the execution, data collection, and manuscript writing. JZ and JL involved in data collection. JW supervised the study and reviewed the manuscript. All authors contributed to the article and approved the submitted version.

## Conflict of Interest

The authors declare that the research was conducted in the absence of any commercial or financial relationships that could be construed as a potential conflict of interest.
